# Tumor Growth Rate Approximation-Assisted Estimation

**Published:** 2007-02-17

**Authors:** Lihua An, S. Ejaz Ahmed, Adnan Ali

**Affiliations:** 1 Department of Mathematics and Statistics, University of Windsor, 401-Sunset Avenue, Windsor, ON N9B 3P4, Canada; 2 Biotechnology Section, Patent Branch/CIPO-Industry Canada, 50-rue Victoria, Gatineau, QC K1A 0C9, Canada

**Keywords:** growth rate, interacting particle system, tumor growth, approximation-assisted estimation, linear and non-linear shrinkage estimators, large-sample bias and risk

## Abstract

From tumor to tumor, there is a great variation in the proportion of cancer cells growing and making daughter cells that ultimately metastasize. The differential growth within a single tumor, however, has not been studied extensively and this may be helpful in predicting the aggressiveness of a particular cancer type. The estimation problem of tumor growth rates from several populations is studied. The baseline growth rate estimator is based on a family of interacting particle system models which generalize the linear birth process as models of tumor growth. These interacting models incorporate the spatial structure of the tumor in such a way that growth slows down in a crowded system. Approximation-assisted estimation strategy is proposed when initial values of rates are known from the previous study. Some alternative estimators are suggested and the relative dominance picture of the proposed estimator to the benchmark estimator is investigated. An over-riding theme of this article is that the suggested estimation method extends its traditional counterpart to non-normal populations and to more realistic cases.

## Introduction

One of the most typical characteristic of malignancy is the disturbance in the balance within cell multiplication. The proliferative activity of the tumor cell population is responsible for the uncontrolled tumor growth. Oncogenic cells are characterized by the continued renewal in their growth and by inhibiting their differentiation. A spatial analysis of the tumor cell growth exhibits a differential rate of growth and may be important in accessing the oncogenic status of the tumor as well as its potential to become malignant.

Braun and Kulperger (1993) and Braun and Kulperger (1995) have introduced an estimator to estimate the growth parameter of an interacting particle system which is discussed in detail by Schürger and Tautu (1976). The interacting particle system theory is also dealt with comprehensively by Liggett (1985) for modeling the proliferation of cells in cancer tumors. They view this interacting particle system as a refinement of the linear birth process which more closely resembles the actual growth of the tumor.

Estimation of the growth rate parameter for linear birth, exponential growth, and Gompertz models has been well-studied. However, the Braun-Kulperger estimator is the first growth rate estimator being proposed for an interacting particle system given the actual tumor data.

The data arises from tumor measurements in mice, for example, at various times following injection of carcinogens. In animal sacrifice experiments, it is only possible to take measurements of the growing tumor at one time point, but several different types of measurements can be taken from the tumor. In longitudinal studies, measurements may be taken at more than one time point, but not as much information can be collected in this case. Usually, only an estimate of tumor volume can be obtained each time. In this paper, we will consider only the former situation. Such data should be considered as coming from an *in vivo* experiment. In particular, we assume that measurements of the total number of cells and the number of boundary cells can be obtained, but at only one time point for each tumor. Boundary cells are defined here as those cells which still have proliferative potential; cells which are in the interior tend to stop proliferating, because of crowding and other effects. Each boundary cell is assumed to split after an exponentially distributed amount of time, with rate λ independent of all other cells, and independent of the history of the process (a Markov assumption).

In this paper, we consider the situation in which the measurements come from different populations. For example, an experimenter may wish to consider data for several populations of animals on different diets, to obtain a potentially more precise estimate for the growth rate. The experimenter is now at risk, since the growth rate may differ depending on the type of diet. A similar situation arises in the case of testing the effectiveness of different radiation treatments on the reduction of tumors, where controlling for the physical presence of the radiation seed is a common practice. Often, the experimenter will conduct a prior experiment to determine if there is such a physical effect by surgically planting a dummy seed in the growing tumor and comparing the resulted growth with a control group which has no seed. Ultimately, the experimenter may want to pool the growth rate estimates from the two populations to obtain a more precise growth rate estimate.

In order to model this type of situation, we suppose that there are *k* possibly different populations of tumors evolving with time and denote the growth rate of the *l*th population by λ*_l_*.

The model is a continuous time Markov chain whose state space is the set of all possible configurations of cells existing at the vertices of a regular lattice *Z^d^*. To each site **x** of the lattice, we associate a set of sites (called the nearest-neighborhood) which is usually of the form:

$$\left\{y:y=x\pm e_{k},\;k=1,2,\ldots ,d\right\}$$

where ± **e***_k_* refers to either the addition or subtraction of the kth standard unit vector (i.e. e*_kj_* = 0, if *j* ≠ *k*, and e*_kk_* = 1).

At the time of exposure to carcinogen, an initial configuration of tumor cells arises from mutation of normal cells. The cells in the initial configuration each waits an independent exponential time, λ*_l_*, before starting fission to produce two offspring. One of the offspring stays at the original site, while the other chooses a site at random from the unoccupied sites of the nearest-neighborhood of the original site. If the nearest-neighborhood is completely occupied, then the new offspring does not survive. In this latter case, we may interpret the cell in the process of fission as hypoxic – cut off from the blood supply by the surrounding layer(s) of cells. The process continues with each of the new offspring waiting and undergoing fission in the same manner.

Braun and Kulperger (1993) have shown that, for a large class of such Markovian models and for *t_f_* > *t_o_*, the growth rate is given by

(1) $$\lambda_{l}=\frac{x_{l}(t_{f})-x_{l}(t_{o})}{\int_{t_{o}}^{t_{f}}b_{l}(t)dt},\;\;\;l=1,2,\cdots k.$$

where *x_l_* (*t*) is the expected number of cells at time *t* and *b_l_* (*t*) is the expected number of boundary cells at time *t* in a tumor from the *lth* population. We let *X_l_*(*t_i_*) be the observed number of cells and *B_l_*(*t_i_*) be the number of boundary cells at time *t_i_*, where *i* = 1, 2,..., *n_l_*, and the *t_i_*’s are assumed to be equally spaced apart. Multiple measurements are required at *t_o_* = *t*_1_ and *t_f_* = *t_n_*. Measurements taken from different animals can be assumed independent, but *B_l_*(*t_i_*) and *X_l_*(*t_i_*) are dependent random variables if taken from the same animal.

If *m_l_* independent observations are available at *t_o_* and *t_f_*, then an estimator of λ*_l_* is given by

(2) $${\hat{\lambda }}_{l}^{B}=2\frac{{\bar{X}}_{l}(t_{f})-{\bar{X}}_{l}(t_{o})}{h_{n}\left(B_{l}(t_{o})+B_{l}(t_{f})+2\sum_{j=2}^{n_{l}-1}B_{l}(t_{j})\right)},$$

where ***X̄****_l_*(*t*) is the sample average of the *m_l_* observations taken at time *t*, and *h_n_* = *t_j_* − *t_j_* _− 1_, *j* = 2,..., *n_l_*. We call this estimator the *baseline* estimator (BE) of the rate parameter λ*_l_*, and use the alternate notation ***λ̂****^B^* = (λ̂_1_*^B^*, λ̂_2_*^B^*, ···, λ̂*_l_^B^*)′.

In the following theorem, we summarize some useful properties of λ̂*_l_^B^* which will form the basis of our asymptotic results.

**Theorem** (Braun and Kulperger (1995)) *Under usual regularity conditions, for each l* = 1, 2,..., *k*,

1. λ̂*_l_^B^* *is a strongly consistent estimator of* λ*_l_*;
2. $\sqrt{m_{l}}({\hat{\lambda }}_{l}^{B}-\lambda_{l})\overset{L}{\to }N(0,\sigma_{l}^{2})$*, as h_n_* → 0, *m_l_* → ∞,

where $\overset{L}{\to }$ means convergence in law and

$$\sigma_{l}^{2}=\lambda_{l}^{2}\frac{Var(X_{l}(t_{f})-X_{l}(t_{o}))}{{\left(x_{l}(t_{f})-x_{l}(t_{o})\right)}^{2}}.$$

We now consider the simultaneous estimation of rate parameter vector **λ** = (λ_1_, λ_2_, ···, λ*_k_*)′ based on random samples of size *m*_1_, *m*_2_,··· *m_k_*, respectively, taken from *k* populations. The main objective of this study is to provide estimators when prior information about the population rates is available, i.e., when it is suspected that **λ** = **λ***^o^*, where {**λ***^o^* = λ_1_*^o^,* λ_2_*^o^,*, ···,λ*_k_^o^*}′ is a vector of the initial valued rates based on previous studies.

Our interest here is to estimate **λ** by combining the sample information and the prior information, i.e., the rates calculated from the sample data and the initial values of the rate parameters. Our goal is to develop natural adaptive estimation methods that are free of subjective choice, tuning parameters, and have superior risk performance under quadratic loss. We demonstrate a well-defined data-based and approximation-assisted shrinkage-type rate estimator that combines estimation problems by shrinking a base estimator to a plausible approximate value. Asymptotic results are demonstrated and the relationship between the base estimator and the family of Stein-rule estimators is discussed. The approximation-assisted estimators are formally defined in section I; meanwhile some preliminary results are stated. In section II, expressions of the asymptotic bias and the asymptotic risk for the estimators of **λ** are presented. In sections III bias and risk analysis is performed and some discussion on how to use these estimators are provided. Section IV summarizes the findings.

## I. Approximation-Assisted Estimation Strategies

In this paper simultaneous estimation of rates from independent Markovian distributions is considered. Assume that *X*_1_, *X*_2_, ···, *X_k_* are independent variables following Markovian models with rate parameters λ*_l_*, *l* = 1, 2, ··, *k*. It is desired to estimate **λ** = (λ_1_, λ_2_, ··, λ*_k_*)′. The baseline estimator **λ̂***^B^* = (λ̂_1_*^B^,* λ̂_2_*^B^,* ··, λ̂*_k_^B^*)′ is based on the respective sample size *m*_1_,···, *m_k_*. The statistical objective is to estimate rate parameter vector **λ** when initial estimates are available from past experiments. Hence, we discuss some approximation-assisted point estimation strategies when (λ_1_, ··, λ*_k_*)′ may be approximated by (λ_1_*^o^***,** ··, λ*_k_^o^*)′.

### Linear Shrinkage Estimator

We first propose a *linear shrinkage estimator (LSE)* of **λ** as follows

(3) $${\hat{\lambda }}^{LS}=\pi \lambda^{o}+(1-\pi ){\hat{\lambda }}^{B}={\hat{\lambda }}^{B}-\pi ({\hat{\lambda }}^{B}-\lambda^{o}),$$

here π ε (0,1) is a coefficient reflecting degree of trust in the prior information about **λ**. If π = 1, we 100% trust the approximation value and hence choose **λ***^o^*; while if π = 0 we do not trust the approximation value at all, and hence choose **λ̂***^B^*- the baseline estimator. Therefore, a value of π near 0 causes **λ̂***^LS^* to be based essentially on the sample data alone. In general **λ̂***^LS^* moves towards **λ̂***^B^* according to the degree of distrust in **λ** = **λ***^o^*. Further, note that **λ̂***^LS^* is a convex-combination of **λ̂***^B^* and **λ** *^o^* via fixed value of π ε (0,1). The value of π may be completely determined by the scientist, depending upon the degree of her/his belief in the initial values. However, it is well documented in literature that estimator like **λ̂***^LS^* has smaller quadratic risk than **λ̂***^B^* in an interval at the expense of poorer performance in the rest of the parameter space induced by the initial values. Not only that, but also the risk function of **λ̂***^LS^* becomes unbounded as the approximation error grows. If the prior information regarding initial values of the parameters is *bad* in the sense that the approximation error is large, the LSE is inferior to **λ̂***^B^*. Alternatively, if the information is *good*, i.e., the approximation error is small, **λ̂***^LS^* offers a substantial gain over **λ̂***^B^*. Nevertheless, in some experimental cases, it is not certain whether or not this information held. Since the information about the parameter is rather uncertain, we incorporate this information using the binary choice estimation.

### Binary Choice Estimator

The binary choice family of estimators is defined as

(4) $${\hat{\lambda }}^{BC}=\{\begin{array}{l} \lambda^{o}\;\;\;\text{if }T<T^{o}\\ {\hat{\lambda }}^{B}\;\;\;\text{otherwise}, \end{array}$$

where *T* is the normalized distance between **λ̂***^B^* and **λ***^o^*, and *T* *^o^* is a specified real number. Further, it can be shown that

(5) $${\hat{\lambda }}^{BC}={\hat{\lambda }}^{B}-I(T<T^{o})({\hat{\lambda }}^{B}-\lambda^{o}),$$

where *I*(*A*) is the indicator of the set *A*. Note that we have replaced π by *I*(*T* < *T* *^o^*) in (3) to obtain (5) with a random dichotomous weight. However, **λ̂***^BC^* has the disadvantage of resulting in extreme outcomes either **λ̂***^B^* or **λ***^o^*. Indeed, if we choose *T* as a suitable test statistic for testing the null hypothesis that **λ** = **λ** *^o^*, then binary choice estimation is generally known as preliminary test estimation. The above insight leads to non-linear shrinkage-type estimation to combine the sample data and past information. This is another basis for combining the information. Stein (1956) demonstrated the inadmissibility of the maximum likelihood estimator (MLE) when estimating the k-variate normal mean vector ***θ*** under quadratic loss. Following this result, James and Stein (1961) and Baranchik (1964) combined the *k*-variate MLE ***θ̂*** with *k*-dimensional fixed null vector, under the normality assumption, as

$${\hat{\theta }}^{S}=(1-c/∥\hat{\theta }-0{∥}^{2})(\hat{\theta }-0),$$

where

0<c<2(k-2),

and demonstrated that for *k* > 2 this estimator dominates the MLE. Further, making use of Stein-type estimator, Sclove and Radhakrishnan (1972) demonstrated the non-optimality of the preliminary test estimation. Hence, here we are confined with Stein-type estimation. However, for *k* < 3, the preliminary test estimation may be a useful choice to tackle the estimation problem at hand.

### Non-linear Shrinkage Estimator

Now using the Stein-like base, we propose the following non-linear shrinkage estimator (NLSE) for the parameter vector, **λ**, as follows:

Define $Y=\sqrt{m^{+}}({\hat{\lambda }}^{B}-\lambda^{\text{o}})$, and

$${\text{S}}_{m^{+}}^{-1}=\text{Diag}\left(\frac{m_{1}}{m^{+}}{\left(\lambda_{1}^{0}{\tilde{\sigma }}_{1}\right)}^{-2},\cdots ,\frac{m_{k}}{m^{+}}{\left(\lambda_{k}^{0}{\tilde{\sigma }}_{k}\right)}^{-2}\right),$$

where

$$\begin{array}{l} {\tilde{\sigma }}^{2}=\frac{S^{2}}{{\left({\bar{X}}_{f}-{\bar{X}}_{o}\right)}^{2}},\\ S^{2}=\frac{\sum_{i=1}^{n}{\left(X_{f}-X_{o}\right)}^{2}}{n-1},\\ m^{+}=\sum_{i=1}^{k}m_{i}. \end{array}$$

The *NLSE* is defined by

$${\hat{\lambda }}^{NS}={\hat{\lambda }}^{B}-(k-3)T^{-1}({\hat{\lambda }}^{B}-{\hat{\lambda }}^{o}),$$

where

(6) $$T={{\text{Y}}^{\prime }\text{S}}_{m^{+}}^{-1}\text{Y},\;\;\;k\geq 4.$$

The estimator **λ̂***^NS^* can be considered as the general form of the shrinkage family of estimators (including linear and non-linear), where the shrinkage of the base estimator **λ̂***^B^* is toward the approximate valued vector **λ***^o^*. Note that the weight in (3) is replaced by a random and smooth function of **λ̂***^B^* and **λ***^o^*, i.e., (*k* − 3)*T*^−1^. However, the proposed **λ̂***^NS^* is no longer a linear function of the benchmark estimator. Further, noting that the shrinkage coefficient (*k* − 3)*T*^−1^ may be greater than 1 causing over-shrinking, we make a truncation that leads to a convex combination of **λ̂***^B^* and **λ***^o^*. This truncated estimator is called *positive-part non-linear shrinkage estimator (PNLSE)*.

### Positive-part Non-linear Shrinkage Estimator

In the spirit of Sclove and Radhakrishnan (1972), the *PNLSE* may be defined as

(7) $${\hat{\lambda }}^{NS+}=\lambda^{o}+{\left[1-(k-3)T^{-1}\right]}^{+}({\hat{\lambda }}^{B}-\lambda^{o}),$$

where [·]^+^ = *max*(0, ·). The positive part estimator is particularly important to control the over-shrinking inherent in **λ̂***^NS^*. The above equation may be rewritten in the following computationally attractive form.

(8) $${\hat{\lambda }}^{NS+}=\lambda^{o}+[1-(k-3)T^{-1}]({\hat{\lambda }}^{B}-\lambda^{o})I(T>k-3)$$

It is interesting to note that the proposed strategy is similar in spirit to the Bayesian model-averaging procedures. However, the main difference is that the Bayesian model-averaging procedures are not optimized with respect to any particular loss function. The present investigation is stimulated by prediction offered by Professor Efron in *RSS News of January, 1995*.

*“The empirical Bayes/James-Stein category was the entry in my list least affected by computer developments. It is ripe for a computer-intensive treatment that brings the substantial benefits of James-Stein estimation to bear on complicated, realistic problems. A side benefit may be at least a partial reconciliation between frequentist and Bayesian perspectives as they apply to statistical practice.”* It may be worth mentioning that this is one of the two areas Professor Efron predicted for continuing research for the early 21st century.

Shrinkage and likelihood-based methods continue to play vital roles in statistical inference. These methods provide extremely useful techniques for combining data from various sources.

## II. Main Results

In this section, we showcase our main results by providing the large-sample expressions for the quadratic bias and risk of the estimators. It is straightforward to show that for large samples, **λ̂***^B^*, **λ̂***^NS^* and **λ̂***^NS^*^+^ are risk equivalent under the non-homegeneity of the parameters. This motivates us to consider a sequence {*C*_(_*_m+_*_)_}

$$C_{\left(m^{+}\right)}:\lambda =\lambda^{\left(m^{+}\right)},$$

where

(9) $$\lambda^{\left(m^{+}\right)}=\lambda +\frac{\delta^{o}}{\sqrt{m^{+}}}.$$

to obtain useful asymptotic results and to provide a meaningful risk performance of the estimators. Note that for **δ***^o^* = **0**, **λ**^(*m*^+)^^ = **λ**, for all *m*^+^.

**Lemma** Under the sequence in (9) and the model assumptions of Section 1, as m^+^ → ∞, $X=\sqrt{m^{+}}({\hat{\lambda }}^{B}-\lambda^{o})$ *follows approximately a multivariate normal distribution with mean vector* ***δ****^o^* *and covariance matrix* **Γ** = *lim* **S**_m+_^−1^*; here we assume that* $lim(\frac{m_{i}}{m^{+}})=\gamma_{i}$.

Now, we present the expressions for the *asymptotic distributional bias* (*ADB*) of the estimators as follows. First, the notation *ψ_k_*(*x*; Δ) stands for the noncentral chi-square distribution function with non-centrality parameter Δ and *k* degrees of freedom. Then we can write $E(\chi_{k}^{-2u}(\Delta ))=\int_{0}^{\infty }x^{-2u}d\psi_{k}(x;\Delta )$.

(10) $$\begin{array}{c} ADB({\hat{\lambda }}^{NS})=-(k-3)\delta E(\chi_{k+1}^{-2}(\Delta )),\\ \Delta =\delta^{\prime }\Gamma^{-1}\delta , \end{array}$$

(11) $$\begin{array}{r} ADB({\hat{\lambda }}^{NS+})=-\delta [\psi_{k+1}(k-3;\Delta )+E\{\chi_{k+1}^{-2}\\ (\Delta )I(\chi_{k+1}^{2}(\Delta ))>(k-3)\}]. \end{array}$$

Now, we transform these functions in a scalar (quadratic) form to obtain a simple yet meaningful interpretation. Define

$$B(.)=[ADB(\hat{\lambda }){]}^{\prime }\Gamma^{-1}[ADB(\hat{\lambda })]$$

as the quadratic bias of **λ̂**. Then

$$\begin{array}{r} B({\hat{\lambda }}^{NS})={\left(k-3\right)}^{2}\Delta {\left[E(\chi_{k+1}^{-2}(\Delta ))\right]}^{2},\\ B({\hat{\lambda }}^{NS+})=\Delta [\psi_{k+1}(k-3;\Delta )+E\{\chi_{k+1}^{-2}\\ (\Delta )I(\chi_{k+1}^{2}(\Delta ))>(k-3)\}{]}^{2}. \end{array}$$

Note that the quadratic bias of **λ̂***^NS^* starts from 0 at Δ = 0, increases to a point, and then decreases towards 0. This is due to the fact that *E*(*χv*^−2^ (Δ))is a decreasing log-convex function of Δ. The behavior of **λ̂***^NS^*^+^ is similar to that of **λ̂***^NS^*. However, the quadratic bias curve of **λ̂***^NS^*^+^ remains below the bias curve of **λ̂***^NS^* for all values of Δ. Note that **λ̂***^B^* is an asymptotically unbiased estimator of **λ**, since it does not incorporate the approximate value, **λ***^o^*, in the estimation process.

To appraise the risk performance of the estimators, we use the quadratic loss function: ℒ(**λ**^⋄^) = *m*^+^(**λ**^⋄^ − **λ**)′**W**(**λ**^⋄^ − **λ**), where **λ**^⋄^ is any estimator of **λ**, and **W** is a positive semi-definite weight matrix. Then, the *quadratic risk* of **λ**^⋄^ is given by

(12) $$R(\lambda^{⋄})=m^{+}E\{(\lambda^{⋄}-\lambda {)}^{\prime }W(\lambda^{⋄}-\lambda )\}.$$

The sequence {C_(_*_m_*_+)_} in (9) will be used to compute the *asymptotic distributional quadratic risk (ADQR)* defined below. First, the *asymptotic distribution function of* $\left\{\sqrt{m^{+}}(\lambda^{⋄}-\lambda )\right\}$ is given by

(13) $$G(z)=\underset{m^{+}\to \infty }{\text{lim}}Pr\{\sqrt{m^{+}}(\lambda^{⋄}-\lambda )\leq z\},$$

for which the limit in (13) exists. Further, define

(14) $$Q=\iint \cdots \int zz^{\prime }dG(z).$$

Finally, the ADQR is defined by *R*(**λ̂**) = trace (**WQ**). Under (9) and the usual regularity conditions, we obtain the ADQR functions of the estimators in the following theorem.

### Theorem

(15) $$R_{1}({\hat{\lambda }}^{B})=trace(W\Gamma ),$$

(16) $$\begin{array}{l} R_{2}({\hat{\lambda }}^{NS})=R_{1}({\hat{\lambda }}^{B})+\delta^{\prime }W\delta (k-3)(k+1)E(\chi_{k+3}^{-4}(\Delta ))\\ -(k-3)R_{1}({\hat{\lambda }}^{B})\{2E(\chi_{k+1}^{-2}(\Delta ))\\ -(k-3)E(\chi_{k+1}^{-4}(\Delta ))\}, \end{array}$$

(17) $$\begin{array}{l} R_{3}({\hat{\lambda }}^{NS+})=R_{2}({\hat{\lambda }}^{NS})-R_{1}({\hat{\lambda }}^{B})E[\{1-\\ \left(k-3)\chi_{k+1}^{-2}(\Delta ){\}}^{2}I(\chi_{k+1}^{2}(\Delta )\leq (k-3)\right]\\ +\delta^{\prime }W\delta [E[2\{1-(k-3)\chi_{k+1}^{-2}(\Delta )\}\\ I(\chi_{k+1}^{2}(\Delta )\leq (k-3)]-E[\{1-\\ \left(k-3)\chi_{k+3}^{-2}(\Delta ){\}}^{2}I(\chi_{k+3}^{2}(\Delta )\leq (k-3)\right]]. \end{array}$$

*Proof.* By Lemma the above relations are obtained using the same arguments as given in Ahmed and Braun (2000).

## III. Risk Performance of the Estimators

The large sample properties of the proposed estimators are discussed in the light of the quadratic loss function. We now investigate the comparative statistical properties of the Stein-type estimators. When *H_o_* is true,

$$\begin{array}{l} R_{1}({\hat{\lambda }}^{B})-R_{2}({\hat{\lambda }}^{NS})=\text{trace}(W\Gamma )\;(k-3)\\ E\left\{2\chi_{k+1}^{-2}-(k-3)\chi_{k+1}^{-4}\right\} \end{array}$$

is a positive quantity. Hence, we conclude that **λ̂***^NS^* dominates **λ̂***^B^* for ***δ*** = **0**. Meanwhile, the maximum risk reduction gain of **λ̂***^NS^* over **λ̂***^B^* is achieved at the null vector. In order to investigate the performance of **λ̂***^NS^* for all values of ***δ***, we characterize a class of positive semi-definite matrices by

(18) $$W^{D}=\left\{\frac{\text{trace}(W\Gamma )}{e_{\max}(W\Gamma )}\geq \frac{k+1}{2}\right\}$$

where *e_max_*(.) means the largest eigenvalue of (.).

**Theorem:** (*Courant*) *If* **A** *and* **B** *are two positive semi-definite matrices with* **B** *nonsingular, both of order* (*q × q*)*, then*

$$e_{min}(AB^{-1})\leq \frac{x^{\prime }\mathrm{Ax}}{x^{\prime }\mathrm{Bx}}\leq e_{max}(AB^{-1})$$

*where e_min_*(.) *means the smallest eigenvalue of* (.) *and* ***x*** *is a column vector of order* (*q* × *1*).

We note that the above lower and upper bounds are equal to the infimum and supremum, respectively, of the ratio $\frac{x^{\prime }\mathrm{Ax}}{x^{\prime }\mathrm{Bx}}$ for **x** ≠ **0**. For **B** = **I**, the ratio is known as the Rayleigh quotient for the matrix **A**. As a consequence of the Courant Theorem,

$$\begin{array}{c} e_{min}(W\Gamma )\leq \frac{\delta^{\prime }W\delta }{\Delta }\leq e_{max}(W\Gamma ),\\ \text{for }\delta \neq 0\;\text{and }W\in W^{D}. \end{array}$$

Thus, under the class of matrices defined in relation (18) we conclude that *R*((**λ̂***^NS^*) ≤ *R*((**λ̂***^B^*) for all ***δ***, where strict inequality holds for some ***δ***. This clearly indicates the asymptotic inadmissibility of (**λ̂***^B^* under local alternatives relative to (**λ̂***^NS^*. The risk of (**λ̂***^NS^* begins with an initial value of 3 and increases monotonically towards trace(**WΓ**). Thus, the risk of (**λ̂***^NS^* is uniformly smaller than (**λ̂***^B^*, where the upper limit is attained when ||***δ||*** → ∞. The result is valid as long as the expectations in (16) exist.

Based on relations (16) and (17), it is seen that

$$R({\hat{\lambda }}^{NS^{+}})/R({\hat{\lambda }}^{NS})\leq 1,\;\;\;\text{for all }\delta ,$$

with strict inequality hold for some ***δ***. Therefore, (**λ̂***^NS^*^+^ asymptotically dominates (**λ̂***^NS^*. Hence, (**λ̂***^NS^*^+^ is superior to (**λ̂***^B^*. The risks of all the estimators depend on the matrices **W** and **Γ**.

### Numerical Risk Output

In order to facilitate numerical computation of the risk functions, we consider the particular case **W** = **Γ**^−1^. In this case trace (**WΓ**) = *k* and ***δ***′**W*δ*** = Δ. The values of the risks are obtained using Maple.

We have numerically computed *R*_1_(**λ̂***^B^*), *R*_2_(**λ̂***^NS^*) and *R*_3_(**λ̂***^NS^*^+^) versus Δ. It is seen that Stein-type estimators dominate **λ̂***^B^* for all the values of Δ. We notice that both estimators have maximum risk gain as compared to **λ̂***^B^* at Δ = 0. In order to quantify this value, the efficiency of the Stein-type estimators relative to **λ̂***^B^* at different values of Δ is computed by using the formula

$$RE_{p}=\frac{R_{1}}{R_{p}},\;\;\;p=2,3.$$

Table 1 provides the estimated relative efficiency of **λ̂***^NS^*^+^ and **λ̂***^NS^* over **λ̂***^B^*, respectively. Both estimators attained maximum efficiency relative ro **λ̂***^B^* at Δ = 0 and the value of the efficiency is a decreasing function of Δ. In addition, table 2 gives the efficiency of **λ̂***^NS^*^+^ relative to **λ̂***^NS^*, i.e., *R*_3_/*R*_2_, for different choices of *k* with Δ = 0, 0.5, 1.0, 1.5 and 3.0.

Seemingly, the magnitude of relative efficiency increases as the value of *k* increases. On the contrary, efficiency decreases with the increasing of Δ.

## IV. Comments and Outlook

The Stein-type estimation strategies are asymptotically superior to strategies based on sample information only. Further, the usual Stein-type estimator is asymptotically dominated by its truncated part. However, we must stress that the important issue here is not the improvement in sense of lowering the risk by using the positive part of the **λ̂***^NS^*. By doing so, **λ̂***^NS^*^+^ removes the over-shrinking behavior of **λ̂***^NS^* when the test statistic takes values near zero. The components of **λ̂***^NS^*^+^ have the same sign as that of components of **λ̂***^B^*. More importantly, positive part estimation provides grounds for studying confidence sets.

In this research we continue the search started four decades ago by Lindley (1962) for new strategies to think about combining estimation problems. In the context of several models, we consider methods for optimally combining the data from various sources. Although the estimation and inference implications of shrinkage estimator are encouraging, some interesting questions remain. For example, we have used the unbiased estimator and the initial value in the proposed estimation methodology. Perhaps one can use biased estimator to further improve the risk-performance of the estimator. Research on the statistical implications of these and other estimators combining possibilities for a range of statistical models is ongoing.

## Figures and Tables

**Table 1:** Relative Efficiency of **λ̂***^NS^*^+^ and **λ̂***^NS^* over **λ̂***^B^* for k = 8.

Delta;	λ̂*^NS^*	λ̂*^NS^*^+^	Delta;	λ̂*^NS^*	λ̂*^NS^*^+^	Delta;	λ̂*^NS^*	λ̂*^NS^*^+^
0.00	1.63	1.71	6.00	1.29	1.31	12.00	1.19	1.19
0.50	1.57	1.65	6.50	1.28	1.30	14.00	1.17	1.17
1.00	1.52	1.60	7.00	1.27	1.28	15.00	1.16	1.16
1.50	1.49	1.55	7.50	1.25	1.27	16.00	1.15	1.15
2.00	1.45	1.51	8.00	1.25	1.26	16.50	1.15	1.15
2.50	1.42	1.48	8.50	1.24	1.25	17.00	1.15	1.15
3.00	1.40	1.44	9.00	1.23	1.24	17.50	1.14	1.14
3.50	1.37	1.42	9.50	1.22	1.23	18.00	1.14	1.14
4.00	1.35	1.39	10.00	1.21	1.22	18.50	1.14	1.14
4.50	1.33	1.37	10.50	1.21	1.21	19.00	1.13	1.13
5.00	1.32	1.35	11.00	1.20	1.21	19.50	1.13	1.13
5.50	1.30	1.33	11.50	1.19	1.20	20.00	1.13	1.13

**Table 2:** Relative Efficiency of **λ̂***^NS^*^+^ over **λ̂***^NS^*.

*k*	Δ = 0	Δ = 0.5	Δ = 1.0.	Δ = 1.5	Δ = 3.0
4	1.320	1.106	1.086	1.070	1.038
5	1.176	1.142	1.116	1.096	1.055
6	1.200	1.162	1.133	1.111	1.066
7	1.216	1.174	1.144	1.121	1.074
8	1.227	1.184	1.152	1.128	1.080
9	1.235	1.190	1.158	1.134	1.084
10	1.242	1.196	1.163	1.138	1.088
11	1.247	1.200	1.167	1.142	1.091
12	1.252	1.204	1.170	1.145	1.094
13	1.256	1.207	1.173	1.147	1.096
14	1.259	1.210	1.175	1.150	1.098
15	1.262	1.212	1.178	1.152	1.100
20	1.273	1.221	1.185	1.159	1.107
25	1.280	1.227	1.190	1.164	1.112
30	1.286	1.231	1.194	1.167	1.116
35	1.289	1.234	1.197	1.170	1.118
40	1.292	1.236	1.199	1.172	1.121
45	1.295	1.238	1.201	1.174	1.122
50	1.297	1.240	1.202	1.175	1.124
